# Ending Tobacco Use Through Interactive Tailored Messaging for Cambodian People With HIV (Project EndIT): Protocol for a Randomized Controlled Trial

**DOI:** 10.2196/48923

**Published:** 2023-06-29

**Authors:** Thanh Cong Bui, Charles E Hoogland, Chhorvann Chhea, Heng Sopheab, Vichea Ouk, Sovannarith Samreth, Bunleng Hor, Jennifer I Vidrine, Michael S Businelle, Ya Chen Tina Shih, Steven K Sutton, Sarah R Jones, Bethany Shorey Fennell, Cherell Cottrell-Daniels, Summer G Frank-Pearce, Chamnab Ngor, Shweta Kulkarni, Damon J Vidrine

**Affiliations:** 1 Department of Family and Preventive Medicine College of Medicine University of Oklahoma Health Sciences Center Oklahoma City, OK United States; 2 TSET Health Promotion Research Center Stephenson Cancer Center University of Oklahoma Health Sciences Center Oklahoma City, OK United States; 3 Department of Health Outcomes and Behavior Moffitt Cancer Center Tampa, FL United States; 4 School of Public Health National Institute of Public Health of Cambodia Phnom Penh Cambodia; 5 National Center for HIV/AIDS, Dermatology and STD of Cambodia Phnom Penh Cambodia; 6 National AIDS Authority of Cambodia Phnom Penh Cambodia; 7 Section of Cancer Economics and Policy, Department of Health Services Research Division of Cancer Prevention and Population Sciences University of Texas MD Anderson Cancer Center Houston, TX United States; 8 Department of Biostatistics and Bioinformatics Moffitt Cancer Center Tampa, FL United States; 9 Department of Biostatistics and Epidemiology Hudson College of Public Health University of Oklahoma Health Sciences Center Oklahoma City, OK United States

**Keywords:** smoking cessation, HIV/AIDS, cost-effectiveness, low- and middle-income countries, Cambodia, Phase-Based Model, RCT, randomized controlled trial, mHealth

## Abstract

**Background:**

The prevalence of smoking remains high in many low- and middle-income countries (LMICs), including the Southeast Asian nation of Cambodia. Smoking is especially hazardous for people with HIV. In Cambodia, approximately 43%-65% of men with HIV and 3%-5% of women with HIV smoke cigarettes. Thus, there is a critical need for cost-effective smoking cessation interventions for Cambodian people with HIV. This paper describes the design, methods, and data analysis plans for a randomized controlled trial assessing the efficacy of a theory-based mobile health smoking cessation intervention in Cambodian people with HIV.

**Objective:**

This 2-group randomized controlled trial compares the efficacy of a mobile health–based automated messaging (AM) intervention versus standard care (SC) in facilitating smoking cessation among Cambodian people with HIV.

**Methods:**

Cambodian people with HIV who currently smoke and are receiving antiretroviral treatment (target, N=800) will be randomized to (1) SC or (2) the AM intervention. SC participants will receive brief advice to quit smoking, written self-help materials, nicotine patches, and will complete weekly app-delivered dietary assessments for 26 weeks. AM participants will receive all SC components (but will complete smoking-related weekly assessments instead of dietary assessments), in addition to a fully automated tailored messaging program driven by the weekly assessments to facilitate smoking cessation. In the Phase-Based Model of smoking cessation, the cessation process is partitioned into 4 phases: motivation, preparation (precessation), cessation (quit date to 2 weeks post quit), and maintenance (up to 6 months post quit). Our AM program targets processes within these phases, including increasing motivation to quit, enhancing self-efficacy, obtaining social support, skills to cope with nicotine withdrawal symptoms and stress, and skills to maintain abstinence. All participants will complete baseline and 3-, 6-, and 12-month in-person follow-up assessments. The primary outcome is biochemically confirmed abstinence at 12 months, with 3- and 6-month abstinence as secondary outcomes. Potential mediators and moderators underlying treatment effects will be explored, and cost-effectiveness will be assessed.

**Results:**

This study was approved by all relevant domestic and international institutional and ethical review boards. Participant recruitment commenced in January 2023. Data collection is expected to conclude by the end of 2025.

**Conclusions:**

By demonstrating the greater efficacy and cost-effectiveness of AM relative to SC, this study has the potential to transform HIV care in Cambodia and prevent tobacco-related diseases. Furthermore, it may be adapted for use in other Cambodian populations and in other low- and middle-income countries. Ultimately, the AM approach to smoking cessation could greatly improve public health in the developing world and beyond.

**Trial Registration:**

ClinicalTrials.gov NCT05746442; https://clinicaltrials.gov/ct2/show/NCT05746442

**International Registered Report Identifier (IRRID):**

PRR1-10.2196/48923

## Introduction

Tobacco use remains the leading cause of preventable morbidity and mortality worldwide [1]. Although the prevalence of tobacco use has declined in high-income nations in recent decades, the prevalence of smoking remains strikingly high in many low- and middle-income countries (LMICs) [2]. In Cambodia, for example, national surveys have indicated that 33%-43% of adult men and 3% of adult women smoke cigarettes [3,4]. Thus, the development and evaluation of sustainable tobacco cessation interventions suitable for widespread implementation in Cambodia are pressing public health needs.

Certain special populations, such as people with HIV, are confronted with disproportionately high tobacco-related health risks. Data from high-income nations indicate that tobacco use represents a leading cause of mortality among people with HIV [5-10]. Nonetheless, there are few efficacious tobacco cessation interventions for people with HIV. The problem of tobacco use among people with HIV appears to be even more critical in Cambodia: available estimates indicate that 43%-65% of men with HIV and 3%-5% of women with HIV smoke cigarettes [11,12]. Although Cambodia has widespread coverage for antiretroviral treatment (ART), no known efforts have been made to provide tobacco treatment to ART recipients who smoke. Thus, complementing ART with efficacious tobacco cessation treatment offers tremendous potential to improve HIV care and prolong life for people with HIV.

This paper describes the design, methods, and analysis plans for an ongoing intervention trial with pharmacological and behavioral treatment components, including a fully automated, smartphone-delivered intervention. Mobile health (mHealth) interventions are proliferating in the United States, but efforts to use similar approaches in Cambodia are extremely limited. The World Health Organization (WHO) acknowledges that the use of mobile and wireless technologies for health promotion is cost-effective, scalable, and sustainable for the least developed countries [13]. In addition, text messaging interventions for smoking cessation have been shown to be effective [14,15], cost-effective [16], and very affordable for tobacco control globally [17-19].

We previously developed the mHealth-based intervention approach discussed in this paper and completed a pilot study with 50 Cambodian people with HIV who smoked and received care at an ART clinic. The results indicated that the biochemically confirmed, 7-day point prevalence abstinence rates at the 2-month follow-up were 40% in the group receiving the mHealth intervention versus 8% in the standard care (SC) control group (relative risk 5.0, 95% CI 1.2-20.5) [20]. This study will evaluate the long-term efficacy of this intervention with a fully powered sample size.

In this study, we are conducting a 2-group randomized controlled trial (RCT) to fully evaluate our mHealth-based automated messaging (AM) intervention versus an SC approach among Cambodian people with HIV who smoke. Specifically, we will assess the long-term (12 months) efficacy of AM, and we will conduct an economic evaluation to compare its cost-effectiveness to that of SC. We will also explore potential mediators and moderators underlying the associations between AM treatment and smoking abstinence (Multimedia Appendix 1).

## Methods

### Ethics Approval

Funding for this study was provided by the National Cancer Institute in 2021 (U01 CA261598-01). It was approved by Moffitt Cancer Center’s institutional review board (IRB; Advarra), which was selected to serve as the single IRB of record for the research described in this protocol by all participating domestic institutions (#00000971), while the IRB of the Ministry of Health of Cambodia—the Cambodian National Ethics Committee for Health Research—approved the research conducted at sites in Cambodia (NECHR #092).

### Study Design

**Figure 1:**
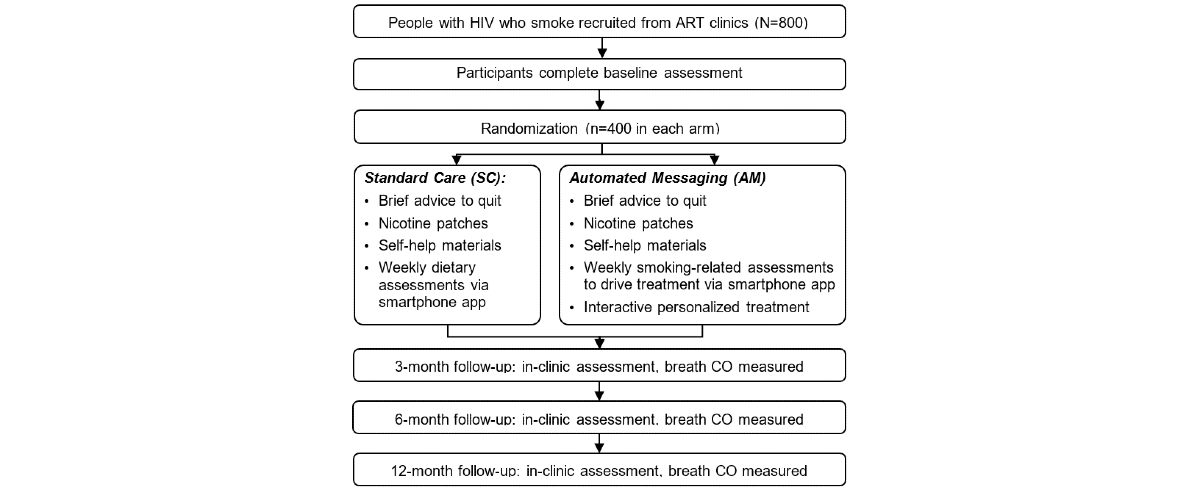
Trial schema. CO: carbon monoxide.

We are using a 2-group RCT to compare the efficacy of 2 smoking cessation interventions: our already-developed smartphone-delivered AM intervention versus SC. Participants are people with HIV recruited through ART clinics in Phnom Penh. Following consent, participants will be randomly assigned to 1 of the 2 study groups: SC (n=400) or AM (n=400). Participants will be asked to complete in-clinic assessments at baseline, and at 3-, 6-, and 12 months following study enrollment (Figure 1). All participants also will complete brief weekly assessments via smartphone. All staff-participant interactions will be conducted to mitigate the potential spread of SARS-CoV-2 and other infections. Should COVID-19–related risks increase in Phnom Penh, we will adapt our procedures by conducting all assessments and follow-ups remotely.

### Conceptual Framework for AM

Our AM intervention is based on the Phase-Based Model (PBM), a theoretical framework that is specific to smoking cessation [21]. The PBM partitions the cessation process into 4 phases: motivation, preparation (precessation), cessation (quit date to 2 weeks post quit), and maintenance (up to 6 months post quit); our project focuses on the 3 latter phases. The PBM helps to identify challenges and opportunities that smokers face at each phase, explains underlying phase-specific mechanisms, and facilitates the selection of intervention components and measures. Several putative mechanisms are relevant across phases and have been empirically shown to be reliably associated with long-term abstinence. These include withdrawal or craving, motivation to quit, positive or negative affect, coping with stress and urges, self-efficacy, and perceived support [21-25]. As such, the AM intervention specifically targets these mechanisms.

### Participants and Eligibility

There are currently 12 ART clinics or hospitals in Phnom Penh that serve approximately 18,800 registered ART patients [26]. We will begin recruitment at sites that serve larger volumes of patients but may add additional clinic sites as needed to reach our accrual goals. Services provided include ART, ART adherence and risk-reduction counseling, nutrition education, and treatments for comorbidities other than tobacco use. Of the ART patients seen at these clinics or hospitals, >90% are of Khmer ethnicity, 49% of them are men, and 93% of them are aged ≥18 years [12,26,27]. The mean levels of education are seventh grade for men and fourth grade for women [28]. The mean duration of ART is 5.7 years. Registered patients at the clinics have periodic prescheduled appointments (about once per month for ART), further facilitating screening and tracking. Study inclusion criteria are: (1) aged ≥18 years; (2) HIV positive; (3) self-report of current combustible cigarette smoking (smoked ≥100 cigarettes in a lifetime and currently smoke ≥1 cigarette per day); (4) willing to set a quit date within 2 weeks of study enrollment; (5) able to provide written informed consent; and (6) able to read Khmer (score ≥4 points on the Rapid Estimate of Adult Literacy in Medicine—Short Form [29]). Exclusion criteria are (1) history of a medical condition that precludes the use of nicotine replacement therapy (NRT), (2) physician or clinician deemed ineligible to participate based on medical or psychiatric condition, or (3) enrolled in another cessation program or use of other cessation medications.

### Baseline Assessment and Randomization

All eligible individuals will be invited to participate. Individuals interested in participating in the study will complete the informed consent process. Enrolled participants will be instructed to complete a tablet-delivered baseline computer-assisted self-interview (CASI), which is managed and delivered by an electronic data capture platform [30-32]. The use of an electronic data capture system that contains programmed logic checks and skip patterns enhances both accurate data collection across sites and timely and secure data transmission. Research staff will help participants complete the assessment if needed. The baseline assessment will take approximately 40 minutes to complete. Enrollees will be randomized to SC or AM via stratified randomization, with sex assigned at birth serving as the stratification variable. All participants will subsequently complete a brief training session on smartphone use and the smartphone app. Smartphones will be loaned to participants who need them. These procedures were followed in our pilot and were found to be feasible in the ART setting.

### Intervention Conditions

#### Standard Care

Participants randomized to SC receive brief advice to quit smoking delivered by trained research staff and self-help materials, including 2 fliers and a 32-page booklet. These self-help materials are from Khmer Quit Now, a national smoking cessation campaign in Cambodia, and are based on the WHO’s “A guide for tobacco users to quit [33].” Participants also receive an 8-week supply of NRT in the form of nicotine patches, which is consistent with US Food and Drug Administration recommendations and has been found to double cessation rates in meta-analyses [34,35]. SC participants complete brief weekly smartphone assessments (4 items) about their diet for a 26-week period. The dietary assessments are included to approximately equate the 2 conditions on the number and frequency of assessments.

#### Automated Messaging

**Figure 2:**
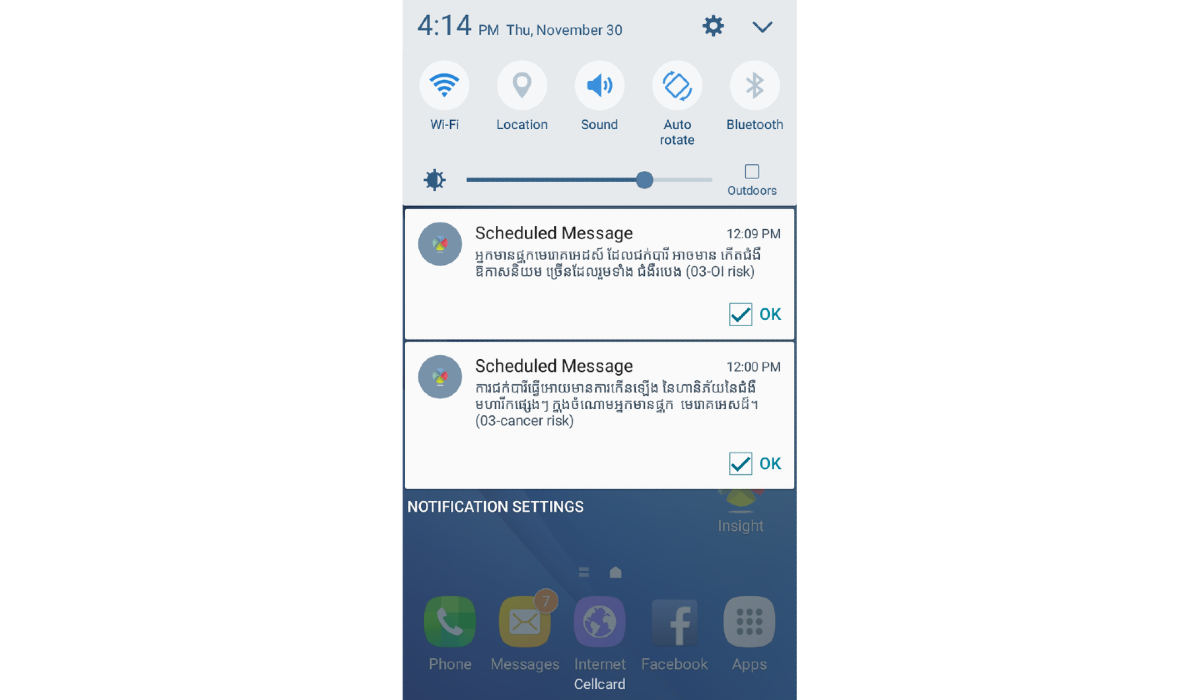
Insight app user interface example.

Participants in the AM group receive the SC components (with the exception of the dietary assessments) plus proactive personalized messages for smoking cessation. The AM content was adapted from the team’s previous efforts and designed to tap the theoretical mechanisms described in the PBM (see Conceptual Framework for AM) [20,36,37]. That is, treatment content is designed to increase motivation, self-efficacy, use of coping skills, and social support and to reduce nicotine withdrawal symptoms and stress. The AM treatment begins immediately after enrollment and continues for a 26-week period. The Insight platform is used to manage and deliver the AM intervention and all weekly assessments [38]. The AM intervention approach allows for several levels of personalization for each participant, as it leverages the adaptive capabilities of the Insight platform. First, at baseline, participants are asked several questions about past quit attempts, preferred coping skills, and the presence or fear of specific health conditions. Messages tailored to these responses are delivered automatically throughout the treatment period. Second, there are different messages for different cessation phases to ensure that the AM intervention targets critical mechanisms of each phase. Phase identification is based on modified PBM definitions; specifically, preparation (enrollment to quit date), cessation (quit date to week 4 post quit date), and maintenance (weeks 4-26 post quit date) [39]. Third, participants are asked to complete brief (4-8 items) smartphone-delivered assessments during each week of the AM treatment course. Some questions vary depending on the phase (eg, current level of intrinsic motivation for preparation or maintenance phases) and other questions are asked in all phases (eg, smoking status in the past week and self-efficacy level). Intervention content (eg, types and frequencies of messages) is based on responses to these weekly assessments and participant phases. Our EndIT-Pilot results demonstrated that the app (Figure 2) worked very well, including being compatible with the 3 largest Cambodian cellular networks, properly delivering messages and weekly assessments, and reliably collecting and transferring data to our encrypted server [20].

### Measures

#### Overview

CASI assessments occur in-clinic at baseline and at 3, 6, and 12 months. Participants also complete brief, weekly assessments via smartphone for the first 6 months of the study. Table 1 summarizes study measures at each assessment.

**Table 1:** Study assessments.

Variable type and measure		Baseline	Weekly^a^	In-clinic follow-up (3, 6, and 12 months)
**Descriptors or potential moderators**				
	Demographics; health literacy [40]	✓		✓^b^
	Alcohol and drug use [41,42]	✓		✓
	CD4 lymphocyte count, viral load, tuberculosis coinfection, COVID-19–related variables, other health outcomes	✓		✓
	Dependence—Heaviness of smoking index [43]	✓		
	Self-report measures of antiretroviral therapy adherence [44]	✓		✓
**Adherence to treatment app**				
	Duration of phone on or off; numbers of messages or images or videos delivered, opened, and marked as viewed; numbers of weekly assessments opened and completed; data syncing frequencies	✓^c^	✓^c^	✓^c^
**Phase-based model mechanisms**				
	Wisconsin Smoking Withdrawal Scale (WSWS-2) [45]	✓^a^		✓^b^
	Reasons for quitting (intrinsic and extrinsic) [46]	✓		✓
	Contemplation Ladder [47,48]	✓	✓	✓
	Kessler Psychological Distress Scale (K6) [49]	✓	✓^b^	✓
	Positive and negative affect schedule (I-PANAS-SF) [50]	✓	✓^b^	✓
	Multidimensional Scale of Perceived Social Support [51]	✓		✓
	Self-Efficacy (related to smoking cessation) [52]	✓	✓^b^	✓
**Economic evaluation**				
	Five-level EQ-5D (EQ-5D-5L; for quality-adjusted life years calculations) [53]	✓		✓
**Primary outcome**				
	Smoking status (includes nicotine replacement therapy adherence, number of quit attempts, days smoking or abstinent, and other products) [54]	✓	✓^b^	✓
	Expired carbon monoxide [54]	✓		✓

^a^Delivered via smartphone app*.*

^b^Brief versions of the scales.

^c^Documented by digital date or time stamps in the smartphone app’s activity log.

#### In-Clinic Assessments at Baseline, 3-, 6-, and 12-Month Follow-Ups

Three forms of data are collected at these time points: CASI, expired carbon monoxide (CO) assessed with a CO monitor (CoVita Breath CO) to verify smoking status biochemically, and medical record data. The CASI assessment is administered at baseline (requires approximately 40 minutes to complete) and at each in-clinic follow-up visit (requires approximately 20 minutes to complete). Medical records are used to collect participants’ clinical information (with participants’ consent), including most recent CD4 counts, viral loads, medication, tuberculosis diagnosis or treatment, and other HIV-related or health conditions. Currently, ART patients at the selected clinics have CD4 lymphocyte count and viral load tests every 3-6 months. However, if participants’ medical records do not contain their CD4 lymphocyte count and viral load test results collected within the last 90 days, participants will be asked to provide a blood sample for these tests, following the current testing protocols at the ART clinics. At the final 12-month follow-up, we also will ask AM participants open-ended questions regarding usability, factors or features that helped them quit, barriers to cessation and how to overcome these barriers, and areas for improvement to the AM treatment program. Participants are compensated US $15 (~60,000 Cambodian riels) for each in-clinic visit.

#### Brief Weekly Smartphone Assessments

Participants are asked to complete brief weekly assessments via smartphones for 6 months, delivered by the Insight app (University of Oklahoma). Participants in AM receive 4-8 questions as described in the Automated Messaging section. Although completion of these brief treatment-driving assessments could be conceptualized as part of the AM treatment, we will attempt to balance the effects of these weekly contacts between the treatment groups. Therefore, participants in SC are also asked to complete a brief 4-item weekly assessment via the app. However, questions for SC participants are about diet. Participants’ responses are encrypted and stored on smartphones and synced to our secure server whenever a connection is active. Thus, our team has near real-time access to responses and we are able to track adherence carefully.

#### Primary Outcome

The primary outcome is smoking status 12 months post enrollment. Abstinence is defined as biochemically confirmed self-reported 7-day point prevalence abstinence with expired CO <6 ppm [54]. Secondary outcomes include 3- and 6-month biochemically confirmed abstinence. We will consider several other common outcomes, such as self-reported abstinence, length of abstinence, and number of quit attempts.

### Data Analysis Plan

#### Aim 1: Conduct an RCT to Evaluate the Efficacy of AM for Cambodian People With HIV Who Smoke

##### H1: At the 12-Month Follow-Up, Smoking Abstinence Will Be Higher in the AM (vs SC) Group

The primary outcome is biochemically verified 7-day point prevalence abstinence at the 12-month follow-up. The primary abstinence analysis will be intention-to-treat, with patients who do not complete the follow-up assessments considered to be smokers; however, we will explore other approaches for dealing with missing data (see Missing Data and Dropouts below).

To estimate the effect of AM on abstinence rates while accounting for the potential clustering of participants recruited from multiple clinic sites, we will use generalized linear mixed models (GLMM) analyses in which intervention groups (AM vs SC) will be estimated as a fixed effect, while the clinic will be modeled as a random effect nested within the treatment condition. Specifically, log binomial mixed models will be used to estimate the relative risk of quitting in the AM (vs SC) group. Although groups should be similar in baseline characteristics due to randomization, we will explore models that control for any demographic or clinical variables that differ between groups at baseline. We will also use GLMM to examine changes in abstinence rates over time while accounting for relevant baseline covariates. Similar GLMM or methodology, as appropriate for each outcome variable, will be used to examine other smoking-related variables, such as continuous abstinence, prolonged abstinence, and quit attempts. Statistical analyses will be performed using SAS (version 9.4; SAS Institute, Inc).

##### Missing Data and Dropouts

Although treating participants lost to follow-up as “not abstinent” has been a widely used analytical strategy in smoking cessation studies, some researchers have pointed to potential problems with this “intent to treat” approach, especially when comparing treatment arms with differential dropout rates [55]. Thus, we will conduct sensitivity analyses to test for treatment differences assuming different missing data mechanisms. For example, we will consider a multiple imputation approach based on smoking-related patient characteristics at baseline as well as demographics to account for potential missing-at-random mechanisms. We also will explore pattern-mixture and selection models to account for potential (and likely) missing-not-at-random mechanisms [56]. Similar findings based on these sensitivity analyses will strengthen our study conclusions.

#### Aim 2: Conduct an Economic Evaluation to Compare the Cost-Effectiveness of the 2 Smoking Cessation Interventions (AM vs SC)

##### H2: AM Will Be More Cost-Effective Than SC

Information on costs and cost-effectiveness is especially important for decision makers in LMICs, as these countries are particularly resource constrained. Evidence of cost-effectiveness is critical for our partners in government agencies to implement and ultimately to scale up the AM program at ART clinics throughout Cambodia. To facilitate decision-making of stakeholders in the public sector, the cost-effectiveness analysis (CEA) will be conducted from the perspective of the governmental service providers.

##### Measures of Costs and Collection of Cost Data

For AM and SC, the costs associated with implementation will come from 4 sources: study personnel, capital costs, production of materials, and distribution of materials. If existing resources are used without charge (eg, office space or computers), we will estimate these costs, given that they would be required for implementation. We will not include the cost of the smartphones because once the efficacy of AM is established, implementation will capitalize on the wide availability of mobile devices in the community and will not involve the distribution of these devices.

##### Measures of Effectiveness

Two commonly used measures of effectiveness, number of quitters and years of life saved (YOLS) [57], will be used to compare with other CEAs of smoking cessation interventions published in the literature. The number of quitters in each arm will be extracted from the 12-month abstinence assessment. We will extrapolate from abstinence to YOLS using a published algorithm that models YOLS per quitter [58]. We will revise the algorithm using more current estimates of age-specific smoking-attributable deaths from tobacco [59], adjusting for reduction in survival for people with HIV. We also will include quality-adjusted life years, which will be calculated based on the five-level EQ-5D (EQ-5D-5L). The EQ-5D-5L is the latest version of the EQ-5D health–related quality of life questionnaire and has been translated into Khmer [53].

##### Analysis

We will summarize findings from the CEA in terms of the incremental cost-effectiveness ratio, calculated as the difference in mean costs between the AM and SC treatments divided by the difference in mean effectiveness between the 2. The incremental cost-effectiveness ratio estimates additional resources needed to achieve an increase in 1 unit of effectiveness and is compared with a commonly published threshold value, which is 3 times the gross domestic product per capita for LMICs according to WHO recommendations [60]. We will assess both short-term and long-term economic impacts of the interventions. To obtain the 95% CIs, we will apply nonparametric bootstrapping methods to the person-level data [61]. We will conduct 1-way sensitivity analyses to examine the impact of alternative measures of cost and outcomes or key assumptions. We will then apply the Bayesian approach to construct the cost-effectiveness acceptability curve and conduct probabilistic sensitivity analysis [62,63]. We will conduct the Bayesian analysis using WinBUGS (BUGS Project), with costs modeled as a gamma or lognormal distribution and abstinence as a binomial distribution [64].

#### Aim 3 (Exploratory): Explore Potential Mediators and Moderators Underlying the Associations Between Treatment Group and Abstinence

We will explore potential mediators and moderators underlying the associations between treatment group and abstinence, as informed by the PBM. We will compare the magnitude of the mediated effects of AM treatment on the outcome of abstinence. Potential mediators include NRT adherence, nicotine withdrawal, motivation, self-efficacy, social support, coping with stress, and positive or negative affect. We also will investigate moderators of the relationship between the treatment group and abstinence to determine the types of participants who may benefit most from AM treatment. We will explore biological sex, HIV stage (as measured by CD4 count and viral load), ART adherence, presence and treatments of common comorbidities (eg, tuberculosis), illicit substance use, and nicotine dependence level as possible moderators. Although we anticipate a small proportion of women in our study, we will make every effort to explore the potential moderating effect of biological sex on the association between treatment arm and abstinence.

## Results

Participant recruitment commenced in January 2023, with all data expected to be collected by the end of 2025. Frequent data analyses to monitor participant performance will occur during the recruitment and follow-up periods, with final data analysis to occur after data collection concludes.

## Discussion

This project capitalizes on a strong, collaborative partnership among researchers at Moffitt Cancer Center, University of Oklahoma Health Sciences Center, The University of Texas MD Anderson Cancer Center, and 3 national governmental agencies that are comprehensively responsible for HIV/AIDS control and care in Cambodia. The investigative team consists of US and Cambodian experts in tobacco, HIV, economic evaluation, mHealth, and health care administration. If our findings indicate that AM is efficacious and cost-effective, our collaboration with influential Cambodian governmental agencies will facilitate wide-scale implementation to HIV clinics across the country. Thus, the project has the potential to transform HIV care delivery throughout the country and to reduce tobacco-induced morbidity and mortality significantly.

Cigarette smoking among people with HIV represents a global public health problem. This issue is even more striking in Cambodia due to a high smoking prevalence in people with HIV and a lack of cessation treatment efforts for this population. Available data (ie, high smartphone ownership rates in Cambodia and high ART coverage) suggest that our approach, which involves recruiting participants from ART clinics and using smartphones to deliver a cessation intervention, represents an ideal and highly feasible way to address this public health problem. Existing data also suggest that Cambodians are receptive to mHealth interventions, and our promising data from the EndIT-Pilot support the feasibility, acceptability, and preliminary efficacy of our approach [20]. This project will provide valuable long-term efficacy data for the AM intervention in a large population of Cambodian people with HIV. Given AM’s potential as a feasible, scalable, highly affordable, and cost-effective standalone intervention, the proposed work has the potential to transform HIV care delivery throughout Cambodia. Sustainability and widespread adoption potential are further enhanced by the direct involvement of Cambodian governmental health agencies. In addition, AM could potentially be integrated with other mHealth services for HIV/AIDS care, such as ART adherence and tuberculosis prevention or treatment. AM also has the potential to be broadly adapted for other populations in Cambodia and other LMICs to address the critical need for tobacco treatment. Finally, very little evidence to support the efficacy and cost-effectiveness of smartphone-based tobacco cessation treatment is available in LMICs [65], and it is largely unknown whether effective cessation interventions from high-income countries are transferable and applicable to LMICs. Therefore, our work will help to answer critical questions including what types of tobacco cessation interventions can be effectively introduced to LMICs and whether this type of mHealth intervention is more cost-effective than alternative treatments in settings with low-cost mobile phone services.

## Appendix

Multimedia Appendix 1 Peer review reports from the National Cancer Institute Special Emphasis Panel on Tobacco Use and HIV in Low and Middle Income Countries.

## Abbreviations

AM: automated messaging
ART: antiretroviral treatment
CASI: computer-assisted self-interview
CEA: cost-effectiveness analysis
CO: carbon monoxide
GLMM: generalized linear mixed model regression
IRB: institutional review board
LMICs: low- and middle-income countries
mHealth: mobile health
NRT: nicotine replacement therapy
PBM: Phase-Based Model
RCT: randomized controlled trial
SC: standard care
WHO: World Health Organization
YOLS: years of life saved
