# Supplementary material for: Ending Tobacco Use Through Interactive Tailored Messaging for Cambodian People With HIV (Project EndIT): Protocol for a Randomized Controlled Trial
Source: JMIR Res Protoc. 2023 Jun 29;12:e48923. doi: 10.2196/48923 (PMC10365624; doi:10.2196/48923)
Supplement: Multimedia Appendix 1 [file resprot_v12i1e48923_app1.pdf]

SUMMARY STATEMENT

(Privileged Communication)

PROGRAM CONTACT:
Mark Parascandola
240-276-6871
paramark@mail.nih.gov

Release Date: 02/03/2021
Revised Date:

Application Number: 1 U01 CA261598-01

Principal Investigators (Listed Alphabetically):

BUI, THANH C
VIDRINE, DAMON J. (Contact)

Applicant Organization: H. LEE MOFFITT CANCER CTR & RES INST

Review Group: ZCA1 SRB-2 (M1)
National Cancer Institute Special Emphasis Panel
Tobacco Use and HIV in Low and Middle Income Countries
AIDS

Meeting Date: 01/13/2021
Council: MAY 2021
Requested Start: 07/01/2021
RFA/PA: CA20-037
PCC: B8GH

Project Title: Ending Tobacco Use through Interactive Tailored Messaging for Cambodian People Living with HIV/AIDS (EndIT)
SRG Action: Impact Score:22
Next Steps: Visit https://grants.nih.gov/grants/next_steps.htm
Human Subjects: 30-Human subjects involved - Certified, no SRG concerns
Animal Subjects: 10-No live vertebrate animals involved for competing appl.
Gender: 1A-Both genders, scientifically acceptable
Minority: 5A-Only foreign subjects, scientifically acceptable
Age: 3A-No children included, scientifically acceptable

Project Year	Direct Costs Requested	Estimated Total Cost
1	498,080	725,690
2	489,015	712,483
3	496,918	723,997
4	496,918	723,997
5	499,856	728,278
TOTAL	2,480,787	3,614,445

1U01CA261598-01 VIDRINE, DAMON

RÉSUMÉ AND SUMMARY OF DISCUSSION: The goal of this application is to test a combination of nicotine replacement therapy (NRT) and a mobile health (mHealth) behavioral intervention to promote smoking cessation in Cambodia for people living with HIV (PLWH) who are also on antiretroviral therapy (ART). This project addresses a highly significant unmet need among Cambodians with HIV, especially males, 43%–65% of whom are smokers (vs. 3%-5% of females). Drs. Damon Vidrine (Moffitt) and Thanh Bui (University of Oklahoma HSC) are both highly qualified investigators with experience in cessation interventions and HIV risk prevention in Cambodia, respectively. The have assembled a very strong team of investigators with expertise in smoking cessation, mHealth interventions, HIV prevention, cost effectiveness and statistical analysis of randomly controlled trials (RCTs). Importantly, the team includes investigators from Cambodia already in a partnership with the MPIs. The main innovative strength of the application is the iterative tailored messaging (in Khmer) that does not require a cell phone network connection. The approach is a 2-arm RCT. It is supported by good pilot data, the intervention is grounded by the Phase-Based Model, abstinence is biochemically verified (exhaled carbon monoxide, eCO), and the automated messaging is participant tailored; these are all noteworthy strengths. The scientific environment for this project is outstanding. However, there are also some minor to moderate weaknesses. Although, justified by the prevalence of smoking in males over females, the number of females in the study may be too limited for analysis. There is little description of the planned data collection, limited involvement with the clinics in Cambodia and description of the interactions with local health officials. There is limited discussion of their dissemination plans and a missed opportunity to collect information about optimizing NRT dosing. Finally, HIV biomarkers as outcomes are not apparent. Nonetheless, this is a very strong application that addresses a critical unmet need in Cambodia and is expected to have a high impact on promoting smoking cessation in Cambodia for people living with PLWH.

DESCRIPTION (provided by applicant): Although the prevalence of tobacco use has declined in developed nations in recent decades, the prevalence of smoking remains strikingly high in many low-income countries. In Cambodia, recent national surveys have indicated that 33%–43% of adult men and 3% of adult women smoke cigarettes. Certain special populations, such as people living with HIV (PLWH), are confronted with disproportionately high risks associated with tobacco use. Available estimates indicate that 43%–65% of male PLWH and 3%–5% of female PLWH smoke cigarettes. Although Cambodia has widespread coverage for antiretroviral treatment (ART), no known efforts have been made to provide tobacco treatment to ART recipients who smoke. Thus, complementing ART with efficacious tobacco cessation treatment offers tremendous potential to improve HIV care and to prolong life for PLWH. To address this need, we propose a randomized controlled trial with pharmacological and behavioral treatment components, including a fully automated, interactive smartphone-delivered intervention. Mobile health (mHealth) interventions are proliferating in the US, but efforts to utilize similar approaches in Cambodia are extremely limited. The World Health Organization acknowledges that mHealth is cost-effective, scalable, and sustainable, including for the least developed countries. We have already developed the mHealth platform and intervention approach proposed for this study, and we have completed a pilot study with 50 Cambodian PLWH who smoked and received care at an ART clinic. Results from this pilot indicated that the biochemically confirmed, 7-day point prevalence abstinence rates at 2-months follow-up were 40% in the group receiving the mHealth intervention vs. 8% in a standard care control group (relative risk: 5.0). In this proposed study, Cambodian PLWH of all sexual identities will be recruited through the 6 largest ART clinics in Phnom Penh, which provide comprehensive care to more than 14,000 PLWH. Participants (n=800) will be randomized to one of 2 treatment groups: Standard Care (SC; n=400), or Automated Messaging (AM; n=400). SC consists of brief advice to quit smoking delivered by research staff, self-help written materials, and an 8-week

supply of nicotine replacement therapy (NRT) in the form of transdermal patches. AM consists of the SC components plus a fully automated smartphone-based treatment program that involves interactive and tailored proactive messaging. The primary outcome is biochemically confirmed self-reported 7-day point prevalence abstinence at 12-months post-study enrollment. Secondary outcomes include abstinence at 3- and 6-months post-enrollment. The primary aims are to evaluate the efficacy of AM for Cambodian PLWH who smoke and to compare the cost- effectiveness of AM vs. SC. If our findings indicate that AM is efficacious and cost-effective, our collaboration with influential Cambodian governmental agencies will facilitate wide-scale implementation to HIV clinics across the country. Thus, the project has the potential to transform HIV care delivery throughout the country and to reduce tobacco-induced cancer morbidity and mortality significantly.

PUBLIC HEALTH RELEVANCE: This project is a collaborative initiative between US researchers and Cambodian public health officials to determine the efficacy of a novel mHealth intervention for smoking cessation among Cambodian people living with HIV/AIDS. The proposed research is relevant to public health because it is ultimately expected to advance understanding of effective tobacco cessation approach in low- and middle-income settings, and subsequently reduce tobacco-induced cancer morbidity and mortality. It is expected that the treatment will be both effective and affordable, thus appropriate for widespread adoption in many resource-poor settings.

CRITIQUE: The written critiques of individual reviewers are provided in essentially unedited form in this section. Please note that critiques and criteria scores, prepared prior to the review meeting, may not have been revised following the meeting.

CRITIQUE 1

Significance: 2
Investigator(s): 2
Innovation: 2
Approach: 2
Environment: 2

Overall Impact:
The proposed study is a 6-site RCT designed to evaluate the impact of standard care (N=400) vs. automated messaging (N=400) to promote 7-day point prevalence abstinence confirmed by eCO at 12 months among PWH in Phnom Penh, Cambodia. Standard of care (SC) involves brief advice to quit smoking, self-help written materials, and 8 week supply of nicotine patches Automated messaging involves SC + fully automated smartphone-based treatment with interactive and tailored messaging. Aims focus on 1. effectiveness to promote tobacco abstinence; 2. cost effectiveness; and 3. Exploring mediators and moderators of the associations between treatment group and abstinence. This application is well written by a strong team of investigators. Strengths of the application include strong pilot data supporting the current application, partnerships with government-level leadership in Cambodia, track record of collaboration among the investigative team members, involvement of advisory group in project development and implementation, and potential for widespread scalability in this setting where treatment options to address tobacco use among PWH are sparse, with relevant cost related-analyses. Moderate weaknesses include limited planned data collection to assess implementation related experiences beyond cost; limited planned inclusion of women; limited consideration of HIV biomarkers/HIV related outcomes as outcomes; and concerns about lack of expressed clinic level support (e.g., letters of support). Overall, despite these considerations, this application has potential for high impact.

1. Significance:
Strengths
- Focus in Cambodia, an area with high need given high prevalence of tobacco use among PWH and lack of treatment.
- High potential for widespread scalability given mobile platform and availability of cellular access and government-affiliated collaborations with advisory board input .
- Rationale is supported by prior pilot study (EndIT-pilot, n=50 PWH) that demonstrated acceptability, feasibility and preliminary efficacy with biochemically confirmed 7-day point prevalence abstinence rates at 2 month follow-up was 40% in group with mHealth intervention vs. 8% in standard care (RR [95% CI]= 5.0 [1.2, 20.5]). Prior to implementation, the intervention was rigorously translated and adapted for the target setting.

Weaknesses
- None noted.

2. Investigator(s):
Strengths
- Dr. Damon Vidrine (PI), Senior Member for the Tobacco Research and Intervention Prevention Program at the H. Lee Moffitt Cancer Center & Research and Vice Chair of the Department of Health Outcomes, and behavior brings strong expertise in tobacco interventions, including among PWH.
- Dr. Bui (MPI), is an Assistant Professor in the Department of Family and Preventive Medicine and member of the Stephenson Cancer Center at OUHSC, brings complementary experiences conducting research in Cambodia among PWH since 2011.
- Dr. Businelle., Scientific Director of the mHealth Shared Resource at the Stephenson Cancer Center, brings strong expertise in mHealth interventions focused on tobacco use and other modifiable cancer risk factors.
- Investigative team has track record of collaboration and complementary expertise (Dr. Jennifer Vidrine [tobacco health risk communication], Dr. Shih [CEA] and Dr. Sutton [statistical analyses]).
- A strong plus to see involvement of public health/HIV leadership in Cambodia as part of the investigative team (Dr. Chhoravann Chhea – Director of National Institute of Public Health; Dr. Heng Sophead – Deputy Director of National Institute of Public Health; Dr. Sun Penh Ly – Director of National Center for HIV/AIDS, Dermatology, and STD, Dr. Leng Bun Hor – Vice Chair of the National AIDS Authority).

Weaknesses
- None noted.

3. Innovation:
Strengths
- Lack of prior studies addressing tobacco use in Cambodia.
- Evaluation of mHealth-based automated messaging with Khmer script.
- Use of flexible Insight™ platform affords bidirectional communication (participants can contact study staff); intervention content and assessments can be done in advance – not real time) and tailored; can be tailored iteratively for the individual; does not require active cellular network connection; and adaptable for post-RCT implementation.

Weaknesses
- Collaborations between government leadership and academics does not seem particularly innovative but is important and enhances potential impact.

4. Approach:
Strengths

- Two arm RCT is appropriate for testing the impact automated messaging (AM) vs. standard care (SC).
- SC includes provision of NRT with patch x 8 weeks + brief advice and self-help materials with dietary assessments.
- AM intervention is theoretically driven and informed by the Phase-Based Model (PBM) to enhance motivation, self-efficacy, use of coping skills, and social support and to reduce nicotine withdrawal symptoms and stress.
- AM is personalized to provide participant-tailored messaging based on assessments.
- Local advisory group to inform project development and implementation.
- Recruitment will occur in 6 of 12 ART clinics/hospitals in Phnom Penh that serve n=14,658 patients and target enrollment appears feasible given smoking prevalence in one of the clinics, acceptability to enrollment, and potential to expand if needed.
- Plan to include adaptive randomization to provide group balance based on biological sex, depression, nicotine dependence, drug use, polytobacco use, reading level and HIV disease severity.
- Evaluation of long-term impact of the 26-week intervention at 12 months is a strength.
- Tobacco abstinence will be confirmed by eCO testing.
- Plan to evaluate mediating and moderating factors of intervention effects.
- Appropriate, clear communication plans including travel of MPI to Cambodia.
- Appropriate recruitment and retention plans.
- Plans for sensitivity analyses to handle dropouts and explore missingness patterns and the impact on outcomes.
- Inclusion of cost and cost effectiveness analyses from the perspective of the government service providers and include determine of incremental cost-effectiveness ratio.

Weaknesses

- Unclear whether lack of demonstrated support for study medical directors of specific clinics is important given other collaborations.
- Although inclusion of NRT as part of SC is a strength, dual NRT is favored over patch alone.
- Exclusion of individuals with smokeless tobacco only (which is more common among women), however, appropriately justified.
- Concern that restriction to patients willing to set quit date limits potential impact.
- HIV biomarkers considered as possible moderators, not evaluated as potential outcomes.

5. Environment:
Strengths

- High need area with limited tobacco treatment interventions in Cambodia for PWH.
- Inclusion of multiple HIV clinical sites enhances generalizability.

Weaknesses

- None noted.

Study Timeline:
Strengths

- Planned activities are appropriate.

Weaknesses

- Timeline lacks adequate details.
- No clear plans for dissemination until phase 3 (Weeks 55-60) of study.

Protections for Human Subjects:
Acceptable Risks and/or Adequate Protections

- Advarra will serve as sIRB.

Data and Safety Monitoring Plan (Applicable for Clinical Trials Only):
Acceptable

- Includes plan for DSMB though members not yet identified.

Inclusion Plans:

- Sex/Gender: Distribution justified scientifically
- Race/Ethnicity: Distribution justified scientifically
- Inclusion/Exclusion Based on Age: Distribution justified scientifically
- The sample will include at maximum estimated 10% women given prevalence of smoked tobacco use.

Applications from Foreign Organizations:
Justified

Resource Sharing Plans:
Acceptable

Budget and Period of Support:
Recommend as Requested

CRITIQUE 2

Significance: 1
Investigator(s): 1
Innovation: 1
Approach: 2
Environment: 1

Overall Impact:
This study investigates using behavioral smoking cessation treatments and NRT (patches) for PLWH in Cambodia. This study proposes an mHealth smart phone intervention though the Insight app, which can be cost effective and could be implemented to other LMICs. There is strong evidence from a pilot study indicating the success that this project could have in increasing rates of smoking abstinence. In this RCT, there are adequate resources an investigator expertise for the study to be successful. Automated messaging in conjunction with NRT has potential to increase smoking cessation efforts in ART clinics in Cambodia. If successful, this study can be replicated in other LMICs. The proposed economic evaluation is also a strength.

1. Significance:
Strengths

- Focuses on the country of Cambodia, which has a very high rate of smoking, particularly for PLWHA.
- The lack of smoking cessation treatments for PLWHA in Cambodia is lacking. Combining ART and smoking cessation is an ideal way to reach this population.
- This project has the potential to be scaled up for other LMICs.

- The collaboration with the Cambodian government has the potential to use ART clinics to deliver smoking cessation to PLWHA.

Weaknesses
- The only worry about this project and the potential of scaling up, is that it could only be done in countries with an adequate supply of NRT.

2. Investigator(s):
Strengths
- This is a very strong team of international researchers focused on HIV, smoking cessation, mHealth, and global health.
- Investigators from Cambodian agencies are included.

Weaknesses
- None noted.

3. Innovation:
Strengths
- The use of the Insight app in Cambodia as a platform to deliver and manage the intervention. This can ultimately be cost effective when managing mHealth interventions. It allows for tailored content with or without a connection, which is important in some LMIC.

Weaknesses
- None noted.

4. Approach:
Strengths
- Use of a randomized control trial to see differences in treatment and control groups, which has been done previously in the US for smoking cessation for PLWHA.
- Based on pilot data with PLWHA that showed potential success for this project.
- Access to over 14,000 potential recruits through ART clinics in Phnom Penh.
- Use of biochemically verified abstinence.
- Use of the Phase-Based Model.
- Inclusion of a local advisory group to aid researchers throughout the study.

Weaknesses
- Sometimes assessments can be viewed as “intervention-y”. It may be that completing once a week assessments without the interactive treatment component would be helpful to know. It seems that this information would be very important when comparing the two groups.

5. Environment:
Strengths
- Excellent environment.

Weaknesses
- None noted.

Study Timeline:
Strengths
- Adequate.

Weaknesses
- None.

Protections for Human Subjects:

Acceptable Risks and/or Adequate Protections
Data and Safety Monitoring Plan (Applicable for Clinical Trials Only):
Acceptable

Inclusion Plans:

- Sex/Gender: Distribution justified scientifically
- Race/Ethnicity: Distribution justified scientifically
- Inclusion/Exclusion Based on Age: Distribution justified scientifically

Applications from Foreign Organizations:
Justified

Select Agents:
Acceptable

Resource Sharing Plans:
Acceptable

Budget and Period of Support:
Recommend as Requested

CRITIQUE 3

Significance:	2
Investigator(s):	2
Innovation:	3
Approach:	2
Environment:	1

Overall Impact:
This project seeks to evaluate an automated mobile technology-based smoking cessation intervention tailored for PLWH in Cambodia. There are few available smoking cessation resources available in Cambodia and the proposed intervention would meet a substantial gap and protect the health of a highly vulnerable population in that country. The intervention will use an existing mobile platform, on which highly promising pilot data are available. The platform allows personalization and tailoring to the target population, with little technical expertise, and has high reach potential. The pairing of the intervention with NRT offers further advantage. Limitations include poor utilization of process data and a vague implementation plan. Overall, the research plan is highly rigorous and a strong investigator team with appropriate experience increases the likelihood of success.

1. Significance:
Strengths

- The project will provide a tailored, personalized, automated smoking cessation intervention for PLWH in Cambodia. There is great need for evidence-based smoking cessation opportunities in Cambodia, and there is very high impact potential among the vulnerable PLWH population.
- Pilot research using the cessation app suggests robust efficacy in the target population.
- The very broach reach or mobile devices and low barriers to utilization of the intervention suggest that implementation has a high likelihood of success.

Weaknesses

- Details on strategies to leverage relationships with Cambodian health officials to promote wider adoption are vague and the potential impact may be overstated.

2. Investigator(s):
Strengths
- An excellent team of investigators representing expertise in tobacco cessation, HIV prevention, tobacco health risk communication, mHealth intervention research, cost effectiveness analysis and RCT statistical evaluation.
- The Co-PIs, Drs. Vidrine and Bui have experience with cessation interventions with PLWH and other underserved populations (Vidrine) and ART adherence and condom use among high HIV risk populations in Cambodia (Bui). Both investigators have experience leading large projects.

Weaknesses
- Collaboration between the Co-PIs appears limited.

3. Innovation:
Strengths
- The project will provide a substantial advance in the adoption of cessation interventions delivered using mobile technology. This has advantages for the smoking cessation field in general but is particularly novel in the Cambodian context, and especially so for PLWH.
- The “Insight” content management system, to be used to tailor and deliver the automated intervention, allows investigators to manage intervention content and collect user-provided data on a continual basis. The capacity to fine-tune the intervention with low technical skill represents a helpful advantage for research and clinical applications, and may support wider future rollout.

Weaknesses
- Smart phone-based cessation interventions are not novel per se and, while some have demonstrated superior efficacy than usual care, they have not necessarily delivered on their promise of a highly scaled, high impact intervention.
- The suggestion that relationships with Cambodian health officials will lead to wider scalability of the proposed intervention is not supported by a formal plan for implementation and is not scientifically innovative.

4. Approach:
Strengths
- Rigorous approach to the study design, incorporating 2-arm RCT, with the intervention consisting of usual best care (including NRT) plus the cessation app. Plans for analysis are sound, including plans to handle missing data & dropouts.
- The intervention is informed by the evidence-guided Phase Based Model, which provided a conceptual framework for the inclusion of specific intervention strategies.
- The study measures are rigorous, including the primary outcome of 12-month biochemically (CO) verified 7-day point prevalence abstinence. The inclusion of HIV clinical data (CD4 count) is an advantage.
- The inclusion of a local advisory group is an advantage. Recruitment plans are realistic with alternative options available.

Weaknesses
- The provision of NRT is a plus, but there is little information about how the app might be used to optimize dosing, resolve problems or request further supplies beyond the standard 8 weeks. This seems like a missed opportunity to fully integrate pharmacotherapy within the app-based intervention.

- While much process data could be collected automatically using the app (including engagement, usability, etc.) there is no formal plan adequately described to collect or analyze data from this important source.

Strengths
- Excellent academic and research environment available at Moffitt Cancer Center and U. Oklahoma.
- Relationships among stakeholder organizations at the study site in Cambodia appear strong and very likely to support productive efforts.

Weaknesses
- None noted.

Study Timeline:
Strengths
- Study timeline is sufficiently detailed and appropriate.

Weaknesses
- None noted.

Protections for Human Subjects:
Acceptable Risks and/or Adequate Protections
- Risks are minimal and been appropriately managed.

Data and Safety Monitoring Plan (Applicable for Clinical Trials Only):
Acceptable
- Robust data safety monitoring plan has been outlined, including plans to institute a DSMB.

Inclusion Plans:
- Sex/Gender: Distribution justified scientifically
- Race/Ethnicity: Distribution justified scientifically
- Inclusion/Exclusion Based on Age: Distribution justified scientifically

Applications from Foreign Organizations:
Justified

Resource Sharing Plans:
Acceptable

Budget and Period of Support:
Recommend as Requested

THE FOLLOWING SECTIONS WERE PREPARED BY THE SCIENTIFIC REVIEW OFFICER TO SUMMARIZE THE OUTCOME OF DISCUSSIONS OF THE REVIEW COMMITTEE, OR REVIEWERS' WRITTEN CRITIQUES, ON THE FOLLOWING ISSUES:

PROTECTIONS FOR HUMAN SUBJECTS (Résumé): ACCEPTABLE

INCLUSION OF WOMEN PLAN (Résumé): ACCEPTABLE

INCLUSION OF MINORITIES PLAN Résumé): ACCEPTABLE

INCLUSION OF BASED ON AGE PLAN (Résumé): ACCEPTABLE

COMMITTEE BUDGET RECOMMENDATIONS: The budget was recommended as requested

---

Footnotes for 1 U01 CA261598-01; PI Name: Vidrine, Damon J.

NIH has modified its policy regarding the receipt of resubmissions (amended applications).See Guide Notice NOT-OD-18-197 at https://grants.nih.gov/grants/guide/notice-files/NOT-OD-18-197.html. The impact/priority score is calculated after discussion of an application by averaging the overall scores (1-9) given by all voting reviewers on the committee and multiplying by 10. The criterion scores are submitted prior to the meeting by the individual reviewers assigned to an application, and are not discussed specifically at the review meeting or calculated into the overall impact score. Some applications also receive a percentile ranking. For details on the review process, see http://grants.nih.gov/grants/peer_review_process.htm#scoring.

MEETING ROSTER

National Cancer Institute Special Emphasis Panel
NATIONAL CANCER INSTITUTE
Tobacco Use and HIV in Low and Middle Income Countries

ZCA1 SRB-2 (M1)
01/13/2021

Notice of NIH Policy to All Applicants: Meeting rosters are provided for information purposes only. Applicant investigators and institutional officials must not communicate directly with study section members about an application before or after the review. Failure to observe this policy will create a serious breach of integrity in the peer review process, and may lead to actions outlined in NOT-OD-14-073 at https://grants.nih.gov/grants/guide/notice-files/NOT-OD-14-073.html and NOT-OD-15-106 at https://grants.nih.gov/grants/guide/notice-files/NOT-OD-15-106.html, including removal of the application from immediate review.

CHAIRPERSON(S)

MUILENBURG, JESSICA L, PHD, MPH
ASSOCIATE PROFESSOR AND GRADUATE COORDINATOR
DEPARTMENT OF HEALTH PROMOTION AND BEHAVIOR
COLLEGE OF PUBLIC HEALTH
UNIVERSITY OF GEORGIA
ATHENS, GA 30602

NORMOLLE, DANIEL P, PHD
ASSOCIATE PROFESSOR
DEPARTMENT OF BIOSTATISTICS
DIRECTOR, UPMC HILLMAN CANCER CENTER
BIOSTATISTICS FACILITY
UPMC HILLMAN CANCER CENTER
PITTSBURGH, PA 15213

MEMBERS

CHOI, WON S, PHD, MPH
PROFESSOR
DEPARTMENT OF PREVENTIVE MEDICINE
AND PUBLIC HEALTH
SCHOOL OF MEDICINE
UNIVERSITY OF KANSAS MEDICAL CENTER
KANSAS CITY, KS 66160

CIOE, PATRICIA ANN, PHD
ASSISTANT PROFESSOR
DEPARTMENT OF BEHAVIORAL AND SOCIAL SCIENCES
BROWN UNIVERSITY SCHOOL OF PUBLIC HEALTH
PROVIDENCE, RI 02912

CUPERTINO, ANAPAULA, PHD
PROFESSOR
DEPARTMENT OF PUBLIC HEALTH SCIENCES
UNIVERSITY OF ROCHESTER
ROCHESTER, NY 14627

EDELMAN, E. JENNIFER, MD, MHS
ASSOCIATE PROFESSOR
DEPARTMENT OF INTERNAL MEDICINE
AND BEHAVIORAL AND SOCIAL SCINECES
YALE UNIVERSITY
NEW HAVEN, CT 06510

FERKETICH, AMY K, PHD, MAS
PROFESSOR
DEPARTMENT OF EPIDEMIOLOGY
COLLEGE OF PUBLIC HEALTH
OHIO STATE UNIVERSITY
COLUMBUS, OH 43210

MIMIAGA, MATTHEW JAMES, MPH, SCD, MA
PROFESSOR
DEPARTMENT OF EPIDEMIOLOGY
FIELDING SCHOOL OF PUBLIC HEALTH
UNIVERSITY OF CALIFORNIA LOS ANGELES
LOS ANGELES, CA 90095

QUINTILIANI, LISA M, PHD
ASSOCIATE PROFESSOR
DEPARTMENT OF MEDICINE
SECTION OF GENERAL INTERNAL MEDICINE
BOSTON UNIVERSITY
BOSTON, MA 02118

REES, VAUGHAN W, PHD
PROFESSOR AND DIRECTOR
DEPARTMENT OF SOCIAL & BEHAVORIAL SCIENCES
CENTER FOR GLOBAL TOBACCO CONTROL
HARVARD SCHOOL OF PUBLIC HEALTH
BOSTON, MA 02115

SCHNOLL, ROBERT A, PHD
ASSOCIATE PROFESSOR, DIRECTOR
DEPARTMENT OF PSYCHIATRY
ABRAMSON CANCER CENTER
PERELMAN SCHOOL OF MEDICINE
UNIVERSITY OF PENNSYLVANIA
PHILADELPHIA, PA 19104

SCIENTIFIC REVIEW OFFICER

SCHWEINFEST, CLIFFORD W, PHD
SCIENTIFIC REVIEW OFFICER
SPECIAL REVIEW BRANCH
DIVISION OF EXTRAMURAL ACTIVITIES
NATIONAL CANCER INSTITUTE- SHADY GROVE
NATIONAL INSTITUTES OF HEALTH
BETHESDA, MD 20892

EXTRAMURAL SUPPORT ASSISTANT

LEE, JULIA Y.
STAFF ASSISTANT
SPECIAL REVIEW BRANCH
DIVISION OF EXTRAMURAL ACTIVITIES
NATIONAL CANCER INSTITUTE SHADY GROVE
NATIONAL INSTITUTES OF HEALTH
ROCKVILLE, MD 20850

Consultants are required to absent themselves from the room during the review of any application if their presence would constitute or appear to constitute a conflict of interest.
